# The Use of Sideline Video Review to Facilitate Management Decisions Following Head Trauma in Super Rugby

**DOI:** 10.1186/s40798-018-0133-4

**Published:** 2018-05-24

**Authors:** Andrew J. Gardner, Ryan Kohler, Warren McDonald, Gordon W. Fuller, Ross Tucker, Michael Makdissi

**Affiliations:** 1 Centre for Stroke and Brain Injury, School of Medicine and Public Health, University of Newcastle, Newcastle, Australia; 2 Hunter New England Local Health District Sports Concussion Program, Newcastle, NSW Australia; 3 HeadSmart™ Sports Concussion Program, Head Office 4 Helensvale Road, Helensvale, Gold Coast, Australia; 4 Australian Rugby Union (ARU), Moore Park, NSW Australia; 5 Sport and Exercise Science, Faculty of Health, University of Canberra, Canberra, ACT Australia; 6 Emergency Medicine Research in Sheffield Group, School of Health and Related Research, University of Sheffield, Sheffield, UK; 7 School of Management Studies, Faculty of Commerce, University of Cape Town, Cape Town, South Africa; 8 World Rugby, Pty (Ltd), Dublin, Ireland; 9 Florey Institute of Neuroscience and Mental Health, Melbourne Brain Centre, Heidelberg, VIC Australia; 10 La Trobe Sport and Exercise Medicine Research Centre, La Trobe University, Bundoora, VIC Australia; 11 Priority Research Centre for Stroke and Brain Injury, Calvary Mater Hospital, Level 5, McAuley Building, Waratah, NSW 2298 Australia

**Keywords:** Rugby, Concussion, Video analysis, In-match concussion management

## Abstract

**Background:**

Sideline video review has been increasingly used to evaluate risk of concussive injury during match play of a number of collision sports, with the view to reducing the incidence of match play concussion injuries. The purpose of this study was to evaluate the effectiveness of sideline video review for identifying and evaluating head impact events in Rugby Union.

**Methods:**

All Australian teams’ 2015 Super Rugby season matches were studied. Meaningful head impact events (HIEs) were identified, comprising events identified and acted upon during matches and events identified through a post-season retrospective review. Video footage of each HIE was coded by two experienced independent sports medicine clinicians to evaluate management decisions made by match-day (MDD) and team doctors (TD). HIE incidences for matches with and without sideline video were compared, and the agreement between game-day video interpretation and the independent clinician opinion calculated.

**Results:**

Seventy HIEs were identified in 83 matches (47 identified during matches and 23 identified post-season), equating to 42.5 HIEs per 1000 player match hours. When video review was available, an unnoticed HIE occurred once every 4.3 matches, compared to once every 2.3 matches when the sideline video review was unavailable. Of the 47 identified in-match HIEs evaluated by TD and MDD during the season, 18 resulted in an immediate and permanent removal, 28 resulted in temporary removal for an off-field assessment, and one resulted in the player continuing the game. Game-day head injury assessment process video decisions agreed with the independent clinician view in 72% of cases, *κ* = 0.49 (95% CI 0.38–0.59, weak agreement).

**Conclusions:**

These findings suggest that access to sideline video review is an important supplementary component to identify potential concussions; however, there is a critical need for improved systems and processes to reduce the likelihood of missing an incident.

## Key Points

- Video review available on the sideline for the match-day doctor (MDD)/team doctor (TD) improves the detection possible HIEs.
- Early post-match video review might be important to pick up ‘missed’ significant HIEs that could be subsequently evaluated on game day by the TD in a timely manner.
- Despite the use of MDDs, TDs, and video feed on the sideline, overt concussion signs can be subtle, or develop later, and so may not be detected during a match.
- The level of agreement between the independent clinician consensus recommendation versus the (MDD) and (TD) in-match decision was weak [*κ* = 0.49 (95% CI 0.38–0.59)].

## Background

Rugby Union is a full-contact collision sport with one of the highest incidences of concussion of all contact sports [1]. At the professional level, concussion is now reported to be the most common match play injury, accounting for approximately one quarter of all injuries [2]. The in-match recognition and management of concussion in full-contact and collision sport is complex and challenging [3]. In the sport of Rugby Union, World Rugby (WR; the international governing body) have a specific regulation for concussion (Regulation 10. Medical [4]) that outlines the ‘recognise and remove’ expectations at all levels of the game [5]. In elite level matches, WR also outlines match-day process and roles for concussion management to reduce the likelihood of missing a concussive incident (see [6, 7] for greater details). The head injury assessment (HIA) process consists of three stages, the first of which (HIA-1) involves the identification of symptoms and/or signs on the field of play, followed by possible removal of a player with an apparent or confirmed concussion for further evaluation. In optimal circumstances, at the elite level, a live pitch-side video feed of the match is also available in multiple angles for the independent match-day doctor (MDD) and team doctor (TD) to immediately review an identified incident to assist with identifying whether Criteria 1 signs necessitating permanent removal are present.

Video analysis has been used to evaluate risk of concussive injury during match play with the view to prevent or reduce the incidence of match play concussion injuries [7–9]. Video review studies have now been conducted in a variety of collision sports such as rugby league [10–13], ice hockey [14], and Australian rules football [9, 15, 16]. The primary aim of this study was to conduct a retrospective video evaluation of in-match head impact incidents (HIE) and HIAs for Australian Super Rugby franchises during the 2015 Super Rugby season and to assess the decision-making process (i.e. permanent removal, remain in play, or return to play) of the MDD and TD. Specific objectives were to (i) describe the epidemiology of HIE in Super Rugby, (ii) evaluate the effectiveness of sideline video review in elite Rugby Union, and (iii) define the inter-rater agreement of video interpretation of HIE.

## Methods

### Setting and Participants

The Super Rugby competition is the elite level state/provincial competition in the southern hemisphere. In 2015, the competition was made up of 15 franchises from Australia (*n* = 5), New Zealand (*n* = 5), and South Africa (*n* = 5). Participants for this study were all Australian Super Rugby franchise players involved in the 2015 Super Rugby season. All players sustaining a meaningful HIE (which was operationalised as sustaining a non-trivial direct blow to the head, or transmission of impulsive force, raising the possibility of a head injury) were included and identified, either by TD or independent MDD, or following post-season retrospective video review. This study was approved by the University of Newcastle Human Ethics Committee (reference no. H-2015-0352).

### Procedures

The WR HIA process provides an advanced care pathway for concussion management at the elite level and consists of three stages delivered by TDs and independent MDDs. The first stage (HIA-1) involves in-match identification and management of meaningful HIEs with the potential to cause concussion. Briefly, players overtly demonstrating signs of concussion (e.g. loss of consciousness, tonic posturing, ataxia) are immediately and permanently removed from play. Where the consequences of a HIE are not clear, players undergo a standardised off-field screening assessment for suspected concussion (HIA-1 screening assessment) [7]. A temporary 10-min substitution is permitted to allow this evaluation. An abnormal screening assessment results in permanent removal from that particular match. Conversely, a normal screening assessment result allows return to the match. Where there are no clinical concerns following an observed HIE, a player will remain in play (i.e. ‘play on’) but may receive ongoing monitoring. At the elite level, a live pitch-side video feed of the match is available in multiple angles for MDD and TDs, with the aid of an independent video operator, to instantly review an observed HIE. This assists with identifying whether signs necessitating immediate and permanent removal are present, whether an off-field screening assessment is indicated, or whether the player can continue with further monitoring. The Hawk-Eye system (Hawk-Eye SMART productions) was used for matches in the current study; however, this was not available at any of the matches played in South Africa during the 2015 season. Live feed, slow motion replay, and bookmarking multiple (upwards of nine) feeds are available. The ability to zoom in and out was contingent upon the broadcast direction to the individual cameramen, not an independent operational function on the sideline. Later stages of the HIA process (HIA-2 and HIA-3) involve serial post-match clinical follow-up and diagnostic assessment for concussion. If a HIE is not detected in match, and a player presents post-match with possible symptoms of potential concussion, they enter the HIA process at stages 2 or 3.

### Data Collection

All MDD/TD’s in-match video and removal from play decision-making was recorded in writing using HIA forms. Data were subsequently stored in a secure World Rugby (WR) database. In addition to the match-day process, WR also have an independent video analyst review all professional matches to identify any potential missed HIE. Separate to the routinely collected WR data, a post-season retrospective video review of all matches involving the five Australian Super Rugby franchises from the 2015 Super Rugby season was conducted by two authors (RK and WM) to identify all possible HIEs. This review was conducted independent of the MDD and/or TD and blinded to the in-match HIA decisions that were made by the MDD and/or TD. Data pertaining to the total number of HIE incidents that did not meet threshold for Criteria 1 or off-field screening criteria was not recorded in this study.

An independent evaluation was then conducted using the broadcaster’s video footage of (i) all MDD/TD-identified in-match HIEs and (ii) all incidents identified in the post-season retrospective video review. This evaluation was conducted by two clinicians (AG and RK) experienced in the video review of concussion. Both experienced clinicians independently provided an opinion on each incident (i.e. immediate and permanent removal; initial removal from play, assessment, but cleared to return to play; or remain in play) based on this video footage. In instances where the management opinions of the two independent clinicians differed, a discussion regarding the decision-making process was conducted to attempt to find agreement. Where it was not possible to reach an agreement, an opinion was obtained from a third clinician (MM), who is also experienced in the on-field management and video review of concussion.

### Analyses

The analysis proceeded in three stages. Firstly, the epidemiology of HIEs in Super Rugby was described, including the number of HIEs detected, and the overall incidence of HIEs. Secondly, HIA process outcomes were described for all included HIEs. Thirdly, the effectiveness of sideline video review for identifying HIEs was evaluated by comparing the number of additional HIEs detected on retrospective post-season review between matches with and without the Hawk-Eye system available. Finally, the agreement between match-day TD/MDD video review decision and the consensus decision from the independent clinicians was compared (i.e. immediate and permanent removal, off-field screening assessment, or remain in play).

Descriptive analyses are presented as frequencies (numbers; *n*) or a percentage of the total. Incidence rates are presented as incidences per 1000 player hours with 95% confidence intervals. Inter-rater agreement analyses used Cohen’s kappa (*κ*) statistics [17] to determine the agreement between the independent raters’ opinions and the MDD/TDs for the three management outcomes. The *κ* coefficients are calculated by considering the proportion of rater agreement and the expected proportion [17]. Using the interpretation of *κ* described by McHugh [17], *κ* agreement was categorised as almost perfect (> 0.90), strong (0.80–0.90), moderate (0.60–0.79), weak (0.40–0.59), minimal (0.21–0.39), and none (0–0.20). A census sample of all HIEs in a single season was analysed. All analysis was performed using IBM SPSS Statistics V.23.0 [18].

## Results

**Fig. 1:**
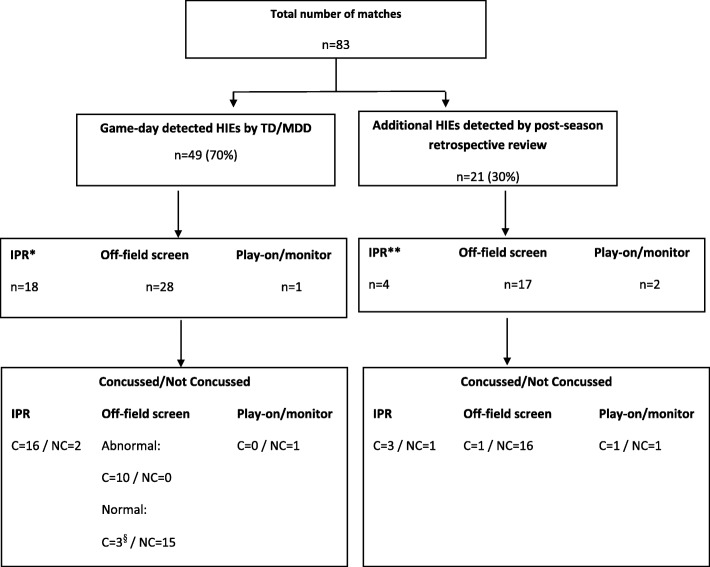
Summary of identified HIEs and diagnosed concussions.*TD/MDD decision; **independent evaluation decision; ^§^players returned to play but were subsequently (post-match) diagnosed with concussion; IPR immediate and permanent removal

There were 83 matches played by the five Australian Super Rugby franchises during the 2015 Super Rugby season. Of these 83 matches, 64 had sideline video review available and 19 matches were played without the use of sideline video. During the 64 matches with sideline video review, 41 HIEs were identified in match and 15 were identified during the post-season retrospective review. In the 19 matches without sideline video review, 6 HIEs were identified in match and 8 were identified post-season. Overall, a total of 47 HIEs were identified in match and 23 HIEs were identified on post-season retrospective review of the match broadcast footage. For the matches reviewed in this study, the World Rugby analyst did not independently identify any additional cases for consideration. The overall incidence of HIEs was 42.3 HIEs per 1000 player match hours (95% CI 32.3–52.0). Overall, there were 34 diagnosed concussions; 25 players suffered one concussion, 3 players suffered two concussions, and one player suffered three concussions during the season. The incidence of diagnosed concussions for the Australian franchises during the 2015 Super Rugby season was 20.5 concussions per 1000 player match hours (95% CI 14.2–28.6). Figure 1 summarises the identified HIEs and concussions.

Of the 47 HIEs identified by the MDD/TD on match day, 18 were deemed to meet Criteria 1 (immediate and permanent removal) requirements and were permanently removed from play. Of these, 16 (89%) were ultimately diagnosed with concussion. Twenty-eight (60%) HIEs were assessed as requiring temporary removal and an HIA-1 off-field screening assessment. In 10 of these cases, the player was permanently removed after an abnormal off-field screen, with all 10 players ultimately diagnosed with concussion. The remaining 18 players were returned to play following normal screening assessments. However, 3 of these players were ultimately diagnosed with concussion. Finally, of the 47 HIEs identified by the MDD/TD on match day, one (2%) player was permitted to play on with further monitoring. Data pertaining to the total number of HIE incidents that did not meet threshold for Criteria 1 or off-field screening criteria was not recorded in this study. Of the 23 HIEs detected on post-season video review, 21 of these players entered stages 2 or 3 of the HIA process and 5 were ultimately diagnosed with concussion. HIA process and concussion outcomes for each HIE are summarised in Fig. 1 and Tables 1 and 2.

**Table 1:** Overview of match-day review decisions

For HIEs identified in season		On-field decision/action			
		Criteria 1	Off-field assessment	Continue to play	All
	Total incidents	18	28	1	47
	Were permanently removed from match	18	10	0	28
	Diagnosed as concussion (permanent removal)	16	10	0	26
	Were returned to match	0	18	1	19
	Diagnosed as concussion (after playing on)		3	0	3
Post-match review recommended action	Agreement with match-day decision	14	19	1	34
	Disagreement with match-day decision	4	9	0	13
	*Should have been Criteria 1*		8	0	8
	*Should have been off-field assessment*	3		0	3
	*Should have been continue to play*	1	1		2

Note. *HIE* head injury event

**Table 2:** Overview of post-season review decisions

	Criteria 1	Off-field assessment	Continue to play	All
Recommendation on post-match video	4	17	2	23
Diagnosed as concussion	3	1	1	5
Total	7	18	3	28

Overall 23 additional ‘missed’ HIEs were detected on the post-season retrospective video review (13.9 HIEs per 1000 player match hours, 95% CI 8.2–19.5). There were 15 missed HIEs in the 64 matches where sideline video review was available (11.7 HIEs per 1000 player match hours (95% CI 5.8–17.6), compared to 6 missed HIEs in 19 matches where sideline video was unavailable (15.8 HIEs per 1000 player match hours, 95% CI 3.2–28.4), incidence rate ratio = 1.8, 95% CI 0.66–4.5, *p* = 0.53*.* Overall, the TD/MDD missed HIEs at a rate of one every 2.3 matches when sideline video was not available, whereas this rate was one every 4.3 matches when sideline video was available (Tables 1 and 2). A summary of the agreement between match-day decisions and the independent evaluation for match-day identified HIEs is shown in Table 3 (*n* = 47). In 14 of the 18 immediate and permanent removal (78%) cases, the consensus opinion of the clinical post-season review agreed with the match-day management decision, whereas in 3/18 (17%) cases, the consensus opinion of the clinical post-match review was that the HIE were considered to meet criteria for an off-field assessment rather than permanent removal, and in 1/18 (6%) cases, the consensus independent evaluation was that incident did not meet criteria for removal and instead the player should have been able to play on with monitoring. In 19 of the 28 HIEs (67%) identified by MDD/TDs as requiring HIA-1 off-field screening assessment, the independent evaluation agreed with the match-day management decision. Whereas in 8/28 (29%) cases, the consensus opinion recommended permanent removal. In 6/8 (75%) of these cases, the HIEs occurred in matches where the MDD/TD did not have access to sideline video to review. In the other two cases, the post-season review recommended permanent removal on the basis of evidence of cervical hypotonia. In 1/28 (4%) case, the independent evaluation recommended play on rather than a HIA-1 temporary removal for the off-field screen.

**Table 3:** Summary of HIEs with and without sideline video access

	With sideline video	Without sideline video	Total/Mean Incidence
Matches	64	19	83
HIEs identified during matches	41	6	47
In-match HIE incidence (per 1000 match hours)	32.0	15.8	28.3
HIEs identified post-season (missed HIEs)	15	8	23
Missed HIE incidence (per 1000 match hours)	11.7	21.1	13.9
Total HIE	56	14	70
Overall HIE incidence (per 1000 match hours)	43.8	36.8	42.2
Rate of missed HIEs (matches per missed HIE)	4.3	2.3	3.6

Note. *HIE* head injury event

The overall inter-rater agreement for the management decisions between the two independent raters (i.e. prior to discussion) was *κ* = 0.88 (95% CI 0.85–0.91), which is considered to be strong agreement [17]. The level of agreement between the independent clinician consensus recommendation versus the MDD/TD in-match decision was *κ* = 0.49 (95% CI 0.38–0.59), which is considered to be weak agreement [17].

## Discussion

To expand previous video analysis work in collision sports, this study explored the use of retrospective video evaluation of HIEs for Australian Super Rugby franchises to assess the live decision-making process of the TD/MDD. Forty-seven HIEs were evaluated by TD and MDD during the season, with a further 23 identified (i.e. a 49% increase in identified HIEs) through a post-season video review process. Of the additional 23 ‘missed’ HIEs, eight occurred in matches without sideline video review (21.1 HIEs per 1000 match hours) and 15 occurred where sideline video was available (11.7 HIEs per 1000 match hours).

Collectively, these findings suggest that access to sideline video review is an important supplementary component to identify potential concussions, since they increase both the number of HIEs identified and the number of HIEs that are subsequently diagnosed as concussions either through meeting the Criteria 1 signs and symptoms for immediate and permanent removal or through indicating an off-field screen that subsequently confirms a concussion (see Table 1). Indeed, there is an 80% relative increase in the rate of missed HIEs when sideline video is unavailable during matches.

Specifically, we found that retrospective analysis to identify all HIEs and to evaluate MDD and TD decisions identified 12 cases where Criteria 1 signs and symptoms indicated immediate and permanent removal. Eight of these HIEs had been identified during matches, but were assessed through the off-field assessment rather than immediate and permanent removal, while four were missed entirely (Table 1). Similarly, 17 cases where a player should have been sent for an off-field assessment were identified through video analysis, but no action was taken. Further cases were identified where a player was immediately and permanently removed when the post-match review suggests that the off-field assessment might have been indicated (*n* = 3) or where the player might have been allowed to play on (*n* = 1). There were three players who were removed from play and diagnosed with concussion post-match after having been returned to play following normal screening assessments. The current study did not examine how or when the diagnosis was made post-match. However, the later stages of the HIA process (HIA-2 and HIA-3), which involves the serial post-match clinical follow-up and diagnostic assessment for concussion are routinely conducted on match day and this may have been when the diagnosis was made. It is unknown whether the players themselves acknowledged they had sustained a concussion despite denying it during the match, if the HIA-2 or HIA-3 assessments detected an abnormal performance, and/or if there was a delay in symptom onset. In addition, in 8/28 (29%) of cases that were considered by the MDD/TD as requiring a HIA-1 off-field screening assessment, and the consensus opinion of the clinicians on post-season review recommended permanent removal based on evidence of cervical hypotonia.

The inter-rater agreement of the post-season review decisions for the management of the identified cases was 0.88 (95% CI 0.85–0.91), strong agreement. The IRA between the MDD/TD game-day management decision and the consensus decision based on the expert reviewer’s post-season review was 0.49 (95% CI 0.38–0.59), weak agreement, suggesting that further education of TD/MDD in the recognition and significance of post-traumatic signs is required.

The addition of sideline video assessment is a further important piece of the puzzle, but there is a critical need for improved systems and processes to reduce the likelihood of missing an incident. In the present study, expert clinicians were able to evaluate the MDD and TD decisions with greater time than is typically the case during matches, as well as to identify HIEs that may have been missed entirely. Such time is unavailable during matches, and so the current results may be viewed as ‘ideal’ scenario changes that video could produce.

However, we do find a significant effect of video on the decisions made regarding removal, off-field screening, and continued play and so suggest that these processes may benefit from the use of ‘spotters’ on game day at various vantage points separate from the TD/MDD but with communication channels to alert the TD/MDD to a potential HIE. Ongoing education of TD/MDD about the early signs of concussion is also a critical point. Furthermore, the use of a post-game video review may be an important method for detecting any ‘missed’ head impact events.

There are a number of ongoing challenges associated with in-match sideline identification of possible concussion, including (i) identification and management of brief early signs (that may have resolved completely by the time that the player is assessed), (ii) the potential evolution of symptoms and signs (including delayed presentation), and (iii) the failure of players to report symptoms on game day. These issues are not mutually exclusive, but the improved education and self-reporting by the player likely has the greatest potential for improving the early identification and on-going management. A growing literature [19] has examined the knowledge and attitude athletes have toward concussion to identify focus areas for education. Commonly, athletes do not consider the concussion they sustained as serious enough to report or to be removed from play and felt they were not in further danger by remaining in play [19]. Asken and colleagues [20] reported that athletes who do not immediately report symptoms of a concussion and continue to participate in athletic activity are at risk for longer recoveries than athletes who immediately report symptoms and are immediately removed from activity, highlighting the importance of early recognition, reporting, and immediate removal of players.

There are a number of limitations associated with this study. Firstly, not all matches had video available on the sideline for the MDD/TD. While this enabled analysis, comparison, and comment on the matches with and without a sideline video review process, it is a limitation to the consistency of the data and generalisability of the results. If video review within professional sports is implemented, then access to high-quality reviews with the capability of multi-angle and slow motion replays to allow for close ups would be optimal [16] and would reduce the likelihood of missing data. The differences in opinion between the MDD/TD management decision and the independent, retrospective evaluation may be attributable to the MDD/TD having access to additional information from video feeds, sideline observation, or on-field interaction with the player, which likely would make their decision more accurate than just using the broadcasters video feed.

Although the video reviewer was blinded to the sideline assessment results and the medical diagnosis of concussion for this study, they were only partially blinded to the use of the HIA. Given that the process for enacting a use of this rule requires the trainer to provide a signal to the sideline, and the official on the sideline identifies the interchange, the video reviewer was able to identify many instances where the HIA was used.

The current study was a post-season review of teams from one of three countries in the tournament in question, and as such, a further limitation of the current study pertains to the generalisability of the current findings to the entire competition, or to other levels of rugby union, or to the modified game (i.e. Rugby 10s or Rugby 7s). Finally, only one reviewer completed the coding of the entire game, for every game in the season that the Australian franchises were involved in; the inter-rater reliability of that type of coding is unknown.

Future studies may focus on the incidence, sensitivity, specificity, positive, and negative predictive power of the concussion signs proposed for the HIA criteria for permanent removal versus an off-field screen. Ascertaining the base rates of these signs during match play will assist in interpreting the video signs on the sideline when reviewing possible HIEs during a match.

## Conclusion

Concussion signs can be subtle, resolve quickly, or become apparent some time after the initial impact [21]. For these reasons, a conservative approach to sideline concussion management is encouraged as the best management strategy and in the best interest of the welfare of the player [22]. However, despite all of the best efforts and intentions, in some instances, some players, who in retrospect should have been removed from play, remain in play. The use of sideline video assessment improves the ability to identify and remove players from play in such cases.

It is important to determine the reliability and validity of identifying the objective signs of concussion when using video analysis because not all instances of specific observed concussion signs occur as a result of the player having sustained a concussion [23] and not all cases of concussion overtly demonstrate signs.

We found a strong agreement between experienced independent post-season video reviewers in the management decision of professional rugby players. The level of agreement between the independent reviewers’ post-season consensus opinion and the MDD/TD in-match decision was, however, weak. Video injury surveillance can be difficult to interpret but may provide a useful adjunct to the clinical assessment of potential concussion. With improved access to video replays and improved communication between video observers and sideline medical personnel, the detection of concussion may improve [23].

## Abbreviations

HIA: Head injury assessment
HIE: Head impact event
IRA: Inter-rater agreement
MDD: Match-day doctor
TD: Team doctor
WR: World Rugby
