# Correction to: The Use of Sideline Video Review to Facilitate Management Decisions Following Head Trauma in Super Rugby

**DOI:** 10.1186/s40798-018-0171-y

**Published:** 2018-12-07

**Authors:** Andrew J. Gardner, Ryan Kohler, Warren McDonald, Gordon W. Fuller, Ross Tucker, Michael Makdissi

**Affiliations:** 1 Centre for Stroke and Brain Injury, School of Medicine and Public Health, University of Newcastle, Newcastle, Australia; 2 Hunter New England Local Health District Sports Concussion Program, Newcastle, NSW Australia; 3 Priority Research Centre for Stroke and Brain Injury, Calvary Mater Hospital, Level 5, McAuley Building, Waratah, NSW 2298 Australia; 4 HeadSmart™ Sports Concussion Program, Head Office 4 Helensvale Road, Helensvale, Gold Coast, Australia; 5 Australian Rugby Union (ARU), Moore Park, NSW Australia; 6 Sport and Exercise Science, Faculty of Health, University of Canberra, Canberra, ACT Australia; 7 Emergency Medicine Research in Sheffield Group, School of Health and Related Research, University of Sheffield, Sheffield, UK; 8 School of Management Studies, Faculty of Commerce, University of Cape Town, Cape Town, South Africa; 9 World Rugby, Pty (Ltd), Dublin, Ireland; 10 Florey Institute of Neuroscience and Mental Health, Melbourne Brain Centre, Heidelberg, VIC Australia; 11 La Trobe Sport and Exercise Medicine Research Centre, La Trobe University, Bundoora, VIC Australia

## Correction

In the original article [1] reference was made to the Hawk-Eye system having been used as the sideline operating system during the 2015 season. This reference was inaccurate; the data included in this study came from either commercial or non-commercial video systems used at the matches played during the 2015 season.

The Hawk-Eye system is mentioned on two occasions in the article:

1. The Procedures section (p2, column 2) states: “The Hawk-Eye system (Hawk-Eye SMART productions) was used for matches in the current study; however, this was not available at any of the matches played in South Africa during the 2015 season.”
  This sentence should read: “Either commercial or non-commercial video systems were used for matches in the current study; however, these were not available at any of the matches played in South Africa during the 2015 season.”
2. The Analyses section (p3, column 1) states: “Thirdly, the effectiveness of sideline video review for identifying HIEs was evaluated by comparing the number of additional HIEs detected on retrospective post-season review between matches with and without the Hawk-Eye system available.”
  This sentence should read: “Thirdly, the effectiveness of sideline video review for identifying HIEs was evaluated by comparing the number of additional HIEs detected on retrospective post-season review between matches with and without the sideline video system available.”

## References

[1] Gardner AJ, Kohler R, McDonald W (2018). The Use of Sideline Video Review to Facilitate Management Decisions Following Head Trauma in Super Rugby. Sports Med – Open.

